# Evaluating the Impact of Different Methods on the Timing and Duration of RSV Epidemics: Analysis of Surveillance Data From the GERi (Global Epidemiology of RSV in Hospitalized and Community Care) Study

**DOI:** 10.1111/irv.70123

**Published:** 2025-06-01

**Authors:** Lisa Staadegaard, Marco Del Riccio, Susanne Heemskerk, Michel Dückers, Rodrigo A. Fasce, Patricia Bustos, Q. Sue Huang, Cheryl Cohen, Jocelyn Moyes, Vernon Jian Ming Lee, Li Wei Ang, Susana Monge, Isabel Martínez‐Pino, Mathieu Bangert, Rolf Kramer, John Paget, Foekje F. Stelma, Jojanneke van Summeren, Saverio Caini

**Affiliations:** ^1^ Netherlands Institute for Health Services Research (Nivel) Utrecht The Netherlands; ^2^ Department of Health Sciences University of Florence Florence Italy; ^3^ Faculty of Behavioral and Social Sciences University of Groningen Groningen The Netherlands; ^4^ Subdepartment of Viral Diseases Public Health Institute of Chile Santiago Chile; ^5^ Respiratory and Exanthematic Virus Section Public Health Institute of Chile Santiago Chile; ^6^ Institute of Environmental Science and Research Wellington New Zealand; ^7^ Centre for Respiratory Disease and Meningitis National Institute for Communicable Diseases Johannesburg South Africa; ^8^ School of Public Health, Faculty of Health Sciences University of the Witwatersrand Johannesburg South Africa; ^9^ Ministry of Health Singapore Singapore; ^10^ National Centre of Epidemiology Institute of Health Carlos III Madrid Spain; ^11^ Public Health Directorate Castilla y León Regional Ministry of Health Valladolid Spain; ^12^ CIBER Epidemiology and Public Health (CIBERESP), Institute of Health Carlos III Madrid Spain; ^13^ Health Economics & Value Assessment Vaccines Sanofi Vaccines Lyon France

**Keywords:** epidemic, estimation method, respiratory syncytial virus

## Abstract

**Background:**

We previously reviewed methods for estimating the timing of respiratory syncytial virus (RSV) epidemics. This study examines the impact of various estimation methods on determining the start, end, duration, and capture rate of RSV epidemics.

**Methods:**

We applied eight estimation methods to RSV surveillance data from the Global Epidemiology of RSV (GERi) study, covering Chile, New Zealand, Singapore, South Africa, Spain, and the United States: 3% and 10% positivity rate, moving epidemic method (MEM), mean positivity, 1.2% total detections, mean and 60% mean number, and 75% average annual percentage (AAP). We compared the median start, end, duration, and capture rate of RSV epidemics obtained from these methods.

**Results:**

Within countries, the median duration of RSV epidemics varied by over 10 weeks, and the median capture rates ranged from > 95 to < 60%, depending on the estimation method. Generally, the 3% positivity rate method identified the longest RSV epidemics (earliest median start and latest end, and highest capture rate). The 10% positivity rate, MEM, and 75% AAP methods indicated the shortest RSV epidemics with the lowest capture rate. The remaining four methods produced intermediate results.

**Conclusions:**

These findings underscore the importance of selecting estimation methods suited to the surveillance system and the intended use, whether for outbreak alert, planning, or targeted interventions.

## 1 Introduction

Respiratory syncytial virus (RSV) is a common cause of acute respiratory infections and is responsible for a substantial burden of disease especially among young children and the elderly [1]. Most children (60–70%) are infected with RSV within their first year of life; while most infections cause mild respiratory symptoms, 2–3% of these result in hospitalization [2]. Risk factors for RSV‐associated hospitalization include prematurity and a history of wheezing [3]. However, the majority of children requiring hospitalization have no underlying medical conditions [4]. Though case‐fatality rates are low in high‐income countries, higher rates have been reported in low‐ and middle‐income countries [4].

In temperate climates, RSV activity is often seasonal, typically peaking in autumn or winter months [5, 6]. Near the equator, RSV activity can be less consistent, with some countries experiencing prolonged or year‐round transmission [6, 7]. Overall, RSV activity appears to follow a latitudinal gradient, with earlier onset in lower latitudes [5]. However, unexplained variations in the timing of RSV activity remain. For example, biennial RSV activity with smaller late epidemics alternating with larger earlier epidemics has been observed in several European countries [7, 8].

Understanding the timing of (sub)national RSV epidemics is increasingly important with the expanded availability of effective prevention and control measures. By November 2024, two monoclonal antibodies (mAbs), Palivizumab (Synagis) and Nirsevimab (Beyfortus) [9, 10], and two vaccines, RSVPreF3 (Arexvy) and RSVpreF (Abrysvo), are available as preventive measures against RSV. Both mAbs are most effective when administered before the onset of RSV epidemic [11]. The vaccines are authorized for individuals aged 60 years and older to protect against severe lower respiratory tract infections (LRTIs) [12, 13]. Furthermore, Abrysvo has been approved by the European Medicines Agency (EMA) for use in pregnant women between 32 and 36 weeks of gestation to protect newborns through 6 months from RSV‐associated LRTIs.

In our previous systematic review, we identified eight estimation methods that are commonly used to determine the start, end, and duration of RSV epidemics [14]. These methods varied in their data requirements (i.e., whether the number of respiratory specimens tested is needed for the denominator), real‐time applicability, and purpose, whether for outbreak alert, planning, or research. In addition to highlighting the broad heterogeneity of assessment methods and their various applications, some studies in the review pointed out that seasonality estimates can vary substantially when different methods are used [15, 16, 17, 18]. This is concerning as the implementation of prevention measures (e.g., the administration of anti‐RSV antibodies and vaccines) depends on accurate seasonality estimates to achieve maximum effectiveness. Here, we build on our previous work and use surveillance data from six countries participating in the Global Epidemiology of RSV (GERi) study to assess the impact of eight estimation methods on the determination of the start and end of RSV epidemics.

## 2 Methods

### 2.1 The Global Epidemiology of RSV Study

The GERi study was launched in 2019 with the aim of improving the understanding of the global epidemiology and timing of RSV epidemics by analyzing surveillance data from countries worldwide [6, 19]. The database of the GERi study consists of surveillance data provided by 16 countries in the Northern and Southern hemispheres and the inter‐tropical belt collected between 2005 and 2019, which includes weekly RSV case counts, number of respiratory samples tested and key surveillance details (e.g., level of care—community or hospitalized, type of surveillance—sentinel or non‐sentinel, case definitions, and laboratory methods) [6, 19]. Throughout this article, we used the term “season” (or “pre‐defined season”) as the period from week 27 to week 26 of the following calendar year for countries in the Northern Hemisphere (Spain and the United States), whereas for countries in the tropics and the Southern Hemisphere, a “season” corresponded to a calendar year and ran from week 1 to week 52 (for “seasons” including a 53rd week, weeks were adjusted backwards, omitting 1 week with no cases).

The RSV surveillance data from Chile, New Zealand, Singapore, South Africa, Spain, and the United States were used in this analysis. For the data from the United States, analyses were conducted separately for each of the 10 US Department of Health and Human Services (HHS) regions (Figure S1) [20], since previous investigations have shown that RSV seasonality differs between regions [21]. These six countries were selected for their geographic representativeness, the diversity in the timing of their RSV epidemics, and the quantity and quality of the available data. Specifically, we included in the analyses only the countries in the GERi database in which ≥ 250 RSV cases were reported in ≥ 5 seasons (once this requirement was met, if for a country there were seasons with < 250 reported RSV cases, these were excluded from the analysis). Additionally, data on the denominator (i.e., tested specimens) was necessary for inclusion, as it was essential for comparing some of the methods we aimed to evaluate. Information on the surveillance system from which the data for each country in this analysis originated can be found in a previous publication from the GERi study [19]. Initially, no further inclusion or exclusion criteria were set, particularly concerning the typical pattern of RSV circulation (e.g., epidemic or year‐round) in the country. Of note, data for New Zealand, Singapore, and South Africa originated entirely from primary care‐based syndromic sentinel surveillance, while for Chile, Spain, and the United States, the totality or part of cases were from hospital‐based RSV surveillance.

### 2.2 Application of Methods

We applied eight quantitative methods identified in our previous review [14]. These methods were selected based on their frequency of application in studies and they fall under six of the eight categories identified in our review. We did not select any method from category IV “Number of detections threshold” because, despite its potential for real‐time use, it is elementary and not well generalizable due to its highly arbitrary threshold selection. We also did not apply the “Change point analysis” (category V) as it typically aims to identify a limited number of changes in the long‐term trend of incidence or mortality rates. Therefore, we considered it less suitable for studying respiratory virus epidemics although it is commonly used in the field of non‐communicable diseases, such as cancer [22].

The eight methods are summarized in Table 1, including data requirements (numerator only or including denominator), the timing of their potential use (retrospective only or also real‐time), and a brief description of how the epidemic was defined within each season. In Spain, surveillance data was available for most seasons only from week 40 to week 20 of the following year; for the calculations, we assumed that no RSV cases occurred during the weeks not covered by surveillance (consequently, the RSV positivity rate was assumed to be 0% during those weeks). The moving epidemic method (MEM) was applied remotely via memapp (http://memapp.iecscyl.com:8080/) following the instructions in the technical manual [23]. In terms of setting, one epidemic wave per season was searched, separately for each country, using cross‐validation and model (non‐manual) optimization. The weekly positivity rate (instead of detections count) was inputted for calculations without any prior transformation. Each method was applied for each season in all the countries included in our study to determine the start and end week, duration of the RSV epidemics, and the corresponding capture rate (defined as the proportion of RSV cases reported between the epidemic's start and end weeks relative to the total number of cases reported during the entire season). Median values across seasons were calculated for each parameter, along with ranges to facilitate comparisons.

**TABLE 1:** Estimation methods that were compared, categorized by data requirement and timing.

Description of the method		Data requirement	Timing	Category^a^
1	Epidemic threshold set at 3% of specimen testing positive for RSV	Denominator	Real‐time (potential)	I: % positivity threshold
2	Epidemic threshold set at 10% of specimen testing positive for RSV			
3	Epidemic located using a specific algorithm and historical data (see text for details)			II: Moving epidemic method (MEM)
4	Epidemic threshold set at the mean percentage of specimen testing positive for RSV in the season^b^ under study		Retrospective	III: Mean positivity threshold
5	Epidemic threshold set at 1.2% of total RSV cases in the season under study	Numerator	Retrospective	VI: % of detections threshold
6	Epidemic threshold set at 60% of the average weekly number of RSV cases in the season under study			VII: Mean detections threshold
7	Epidemic threshold set at the average weekly number of RSV cases in the season under study			
8	Shortest stretch of consecutive weeks accounting for at least 75% of all RSV cases in a season			VIII: Average annual percentage (AAP)

^a^ The category number corresponds to that in Staadegaard et al. 2024 [*Staadegaard 2024*]. As detailed in the text (Methods), seasonality methods falling in categories IV (“Number of detections threshold”) and V (“Change point analysis”) were not considered in the present analysis.

^b^ As detailed in the text (Methods), the term “season” refers to the period from week 27 to week 26 of the following calendar year for countries in the Northern Hemisphere, or to the calendar year (weeks 1 to 52) for countries in the tropics and the Southern Hemisphere.

## 3 Results

We analyzed surveillance data comprising 441,490 RSV cases, with 79.0% originating from the United States. Across the six countries, the overall positivity rate ranged from 7.1% in the HHS region 2 (New York) in the United States to 16.2% in Chile (Table 2). In Spain (Figure S2) and the United States (Figure S3), the total number of RSV cases detected per a season tended to increase over time.

**TABLE 2:** Countries and seasons included in the analysis, number of RSV detections and processed respiratory specimens (total included and range by season), and % positivity rate (overall and range by season).

Country	Seasons	RSV detections, *N* (total, range)	Specimens, *N* (total, range)	Positivity rate, % (overall, range)
***Chile***	*N* = 7 (2012–2018)	36,835 (4727‐6513)	228,015 (31,039‐33,516)	16.0% (14.2–20.2%)
***New Zealand***	*N* = 7 (2012–2018)	2451 (422–715)	29,866 (1962‐5592)	13.4% (11.3–22‐4%)
***Singapore***	*N* = 8 (2011–2018)	14,374 (1574‐2250)	151,158 (16,730‐22,122)	9.5% (8.2–10.7%)
***South Africa***	*N* = 10 (2009–2018)	6690 (280–1116)	51,745 (3332‐8071)	12.9% (6.2–16.8%)
***Spain***	*N* = 13 (2006/2007–2018/2019)	30,688 (755–4339)	214,713 (3647‐37,715)	14.3% (11.4–20.9%)
***United States***				
HHS region 1 (Boston)	*N* = 10 (2009/2010–2018‐2019)	13,333 (359–3054)	177,249 (3041‐40,932)	7.5% (5.7–16.2%)
HHS region 2 (New York)	*N* = 10 (2009/2010–2018‐2019)	32,464 (1394‐5211)	455,114 (14,086‐94,139)	7.1% (5.5–10.1%)
HHS region 3 (Philadelphia)	*N* = 12 (2007/2008–2018‐2019)	25,477 (611–4208)	330,395 (6348‐57,287)	7.7% (5.8–11.3%)
HHS region 4 (Atlanta)	*N* = 10 (2009/2010–2018‐2019)	29,623 (794–5193)	413,969 (10,353‐86,300)	7.2% (6.0–10.4%)
HHS region 5 (Chicago)	*N* = 14 (2005/2006–2018‐2019)	77,036 (250–14,018)	1,038,458 (1800‐207,811)	7.4% (5.5–14.9%)
HHS region 6 (Dallas)	*N* = 10 (2009/2010–2018‐2019)	55,412 (529–12,234)	609,370 (10,304‐130,976)	9.1% (5.1–10.9%)
HHS region 7 (Kansas City)	N = 7 (2012/2013–2018‐2019)	13,509 (967–3174)	176,353 (9538‐34,673)	7.7% (5.9–10.1%)
HHS region 8 (Denver)	*N* = 11 (2008/2009–2018‐2019)	36,192 (331–5781)	399,016 (6576‐60,757)	9.1% (4.3–15.5%)
HHS region 9 (San Francisco)	*N* = 9 (2010/2011–2018‐2019)	41,895 (486–11,027)	564,107 (11,455‐130,272)	7.4% (3.0–9.8%)
HHS region 10 (Seattle)	*N* = 10 (2009/2010–2018‐2019)	23,953 (322–5531)	302,755 (6401‐78,365)	7.9% (5.0–10.1%)

**FIGURE 1:**
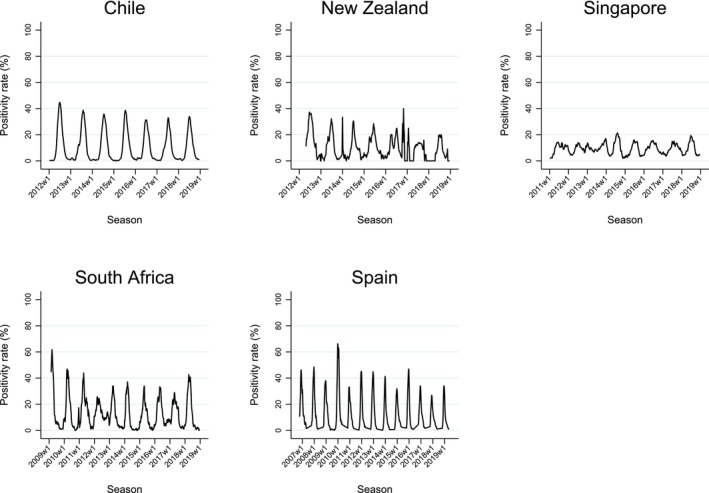
Seasonality of RSV by country: 3‐week moving average positivity rate in Chile, New Zealand, Singapore, South Africa, and Spain.

**FIGURE 2:**
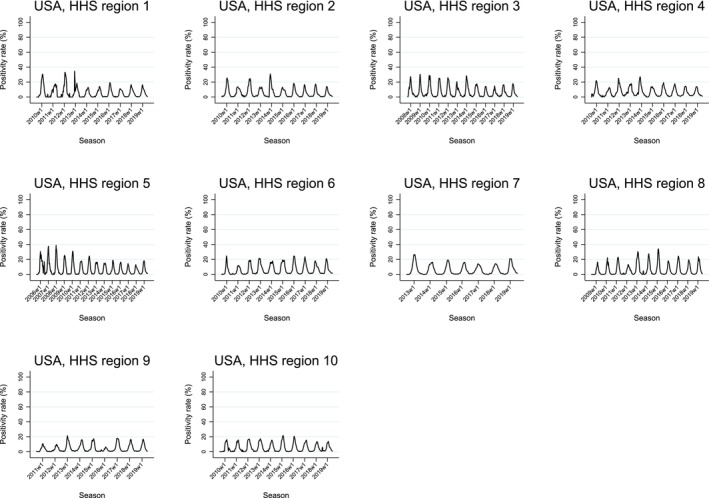
Seasonality of RSV by country: 3‐week moving average positivity rate in the United States by HHS region.

Figure 1 shows the time series of RSV positivity rate (3‐week moving average) for Chile, New Zealand, Singapore, South Africa, and Spain, and Figure 2 illustrates the trend for the United States by HHS region. In Chile, Spain, and most US regions, a single distinct epidemic peak was evident, followed by periods of minimal or no activity. New Zealand's data shows more variability. In South Africa, periods of increased RSV activity were interspersed with intervals of low or no activity, though this pattern was less stable than in Chile and Spain. In Singapore, RSV activity was observed nearly year‐round, with higher RSV cases bridging consecutive years—a pattern often observed in tropical regions. Given Singapore's near‐continuous RSV activity, questions regarding the start, end, and duration of epidemic periods are less relevant; hence, Singapore was excluded from subsequent analyses.

### 3.1 Comparison of Seasonality Methods

The median start and end weeks, duration, and capture rate (with range) obtained by each estimation method are summarized in Table 3 (Chile, New Zealand, South Africa, and Spain) and Table 4 (10 US HHS regions). The *3% positivity* method consistently marked the earliest start and latest end week, resulting in the longest RSV epidemic duration and the highest capture rate among the methods. Specifically, RSV epidemic durations using the 3% positivity method reached a median of 21 weeks (approximately 5 months) or longer across all countries and US regions, with maximum durations of 35 weeks (approximately 8.5 months) in South Africa and 32 weeks (≈8 months) in Spain. The median capture rate ranged from 93.3% in US HHS region 9 to over 98% in New Zealand, South Africa, and Spain. On the other hand, AAP and MEM generally produced the shortest duration of RSV epidemics, whereas in the United States, the 10% positivity method typically produced the shortest epidemics. When comparing methods, the median difference in epidemic durations was as high as 22 weeks (approximately 5.5 months) in Spain.

**TABLE 3:** Seasonality parameters (start and end weeks, duration, and capture rate) obtained by applying various estimation methods in Chile, New Zealand, South Africa, and Spain (median and range are reported).

Country	Seasonality parameter (median, range)	Estimation method (category)							
		3% Positivity (I)	10% Positivity (I)	MEM (II)	Mean positivity (III)	1.2% Total detections (VI)	60% Mean number (VII)	Mean number (VII)	75% AAP (VII)
Chile	Start week	***17 (13–20)***	19 (18–23)	22 (20–24)	20 (18–23)	20 (18–23)	20 (18–22)	21 (21–23)	*23 (23–25)*
	End week	***42 (40–45)***	38 (36–39)	35 (34–38)	38 (36–39)	37 (36–41)	38 (36–41)	36 (35–39)	*34 (32–36)*
	Duration (weeks)	***26 (25–30)***	19 (17–21)	15 (14–15)	19 (17–21)	19 (17–19)	19 (18–20)	16 (15–17)	*12 (10–12)*
	Capture rate (%)	***97.7 (96.7–98.5)***	91.7 (89.7–95.8)	82.3 (79.4–87.4)	91.7 (89.7–94.3)	91.9 (90.5–94.3)	92.8 (91.2–94.3)	89.0 (84.5–89.8)	*76.3 (75.7–78.7)*
New Zealand	Start week	***12 (1–18)***	18 (11–22)	21 (15–24)	18 (9–19)	19 (17–21)	18 (12–19)	19 (17–21)	*24 (20–25)*
	End week	***42 (38–45)***	*33 (32–38)*	*33 (29–38)*	37 (32–43)	37 (35–39)	39 (36–43)	38 (36–39)	35 (32–36)
	Duration (weeks)	***28 (24–42)***	19 (12–22)	13 (10–17)	23 (19–28)	20 (18–21)	24 (22–27)	20 (18–21)	*12 (12–15)*
	Capture rate (%)	***98.5 (96.9–100)***	86.8 (76.1–93.4)	*76.1 (50.9–81.7)*	93.2 (85.7–98.3)	92.5 (87.4–97.8)	97.2 (93.3–99.0)	92.7 (87.4–97.8)	76.8 (75.0–78.0)
South Africa	Start week	***2 (1–7)***	5 (1–8)	*8 (5–11)*	6 (2–8)	6 (3–8)	6 (3–8)	7 (5–14)	*8 (5–11)*
	End week	***35 (20–52)***	27 (19–35)	*20 (12–26)*	25 (18–30)	29 (18–48)	29 (18–48)	27 (17–30)	27 (16–38)
	Duration (weeks)	***35 (19–52)***	23 (15–33)	*14 (4–18)*	20 (12–24)	23 (12–44)	24 (12–44)	19 (11–22)	19 (11–34)
	Capture rate (%)	***98.6 (93.8–99.6)***	85.2 (78.5–91.2)	*59.7 (10.5–77.8)*	84.0 (47.8–89.7)	87.9 (75.5–93.5)	88.1 (75.5–93.5)	78.2 (51.3–89.7)	75.6 (75.1–77.8)
Spain	Start week	***43 (41–45)***	46 (42–47)	46 (42–48)	45 (42–48)	45 (42–47)	45 (42–47)	46 (42–48)	*48 (44–50)*
	End week	***13 (8–20)***	7 (4–11)	6 (3–8)	9 (5–11)	10 (8–13)	10 (8–13)	8 (6–10)	*5 (3–8)*
	Duration (weeks)	***32 (18–28)***	15 (10–17)	13 (11–16)	16 (13–20)	18 (15–21)	19 (15–21)	15 (13–19)	*10 (9–13)*
	Capture rate (%)	***98.2 (93.8–99.3)***	88.8 (73.2–94.5)	80.1 (73.2–87.6)	89.5 (84.0–95.7)	94.4 (91.0–97.1)	94.6 (91.0–97.1)	90.4 (87.9–93.4)	*76.5 (75.1–78.3)*

*Note:* In each country, we highlighted the earliest start and latest end weeks, longest duration, and highest capture rate (median values) in bold, and the latest start and earliest end weeks, shortest duration, and lowest capture rate (median values) in italics.

**TABLE 4:** Seasonality parameters (start and end weeks, duration, and capture rate) obtained by applying various estimation methods in each of the 10 HHS regions in the United States (median and range are reported).

Country	Seasonality parameter (median, range)	Estimation method (category)							
		3% Positivity (I)	10% Positivity (I)	MEM (II)	3% Positivity (I)	1.2% Total detections (VI)	60% Mean number (VII)	3% Positivity (I)	75% AAP (VII)
HHS 1	Start week	***46 (42–50)***	*52 (48–7)*	47 (50–4)	49 (46–3)	50 (44–3)	49 (44–3)	50 (45–4)	*52 (48–4)*
	End week	***17 (15–20)***	*11 (4–19)*	13 (10–19)	14 (12–19)	16 (12–19)	***17 (12–19)***	14 (11–17)	12 (8–15)
	Duration (weeks)	***23 (21–28)***	*9 (6–16)*	16 (14–18)	19 (14–21)	21 (14–22)	21 (14–22)	17 (12–20)	13 (9–14)
	Capture rate (%)	***95.6 (93.9–99.7)***	*56.8 (32.7–91.1)*	83.7 (74.5–92.5)	91.5 (84.0–97.8)	93.5 (88.9–96.4)	93.6 (88.9–96.4)	89.2 (83.3–93.6)	77.5 (75.1–78.5)
HHS 2	Start week	***43 (41–45)***	*47 (44–50)*	*47 (44–49)*	45 (43–48)	45 (43–48)	45 (43–48)	46 (45–49)	*47 (46–50)*
	End week	11 (15–18)	*7 (1–10)*	9 (4–13)	11 (5–15)	12 (9–17)	***14 (9–18)***	11 (8–15)	9 (4–12)
	Duration (weeks)	***23 (21–28)***	*12 (8–17)*	14 (12–19)	18 (15–22)	20 (18–23)	22 (18–24)	18 (16–20)	14 (11–15)
	Capture rate (%)	***95.4 (88.8–96.8)***	*65.4 (45.1–90.3)*	73.3 (61.7–85.2)	87.7 (76.1–92.3)	91.5 (90.7–93.7)	92.8 (91.0–94.9)	88.2 (86.2–89.2)	76.5 (75.2–78.3)
HHS 3	Start week	***44 (40–49)***	*48 (43–52)*	*48 (43–51)*	46 (43–50)	46 (40–50)	45 (40–49)	47 (43–51)	*48 (43–1)*
	End week	***16 (13–19)***	*8 (1–13)*	9 (4–13)	11 (8–15)	14 (11–16)	14 (11–16)	11 (8–14)	9 (7–12)
	Duration (weeks)	***25 (19–30)***	*13 (5–17)*	14 (12–17)	18 (13–22)	21 (18–25)	22 (19–25)	17 (15–20)	14 (12–17)
	Capture rate (%)	***94.3 (90.7–96.7)***	73.6 (34.2–85.7)	*73.0 (59.4–84.8)*	87.6 (74.3–92.8)	91.5 (86.2–92.9)	92.3 (88.7–93.8)	85.1 (81.3–88.7)	77.2 (75.3–78.2)
HHS 4	Start week	***39 (31–44)***	*45 (41–7)*	*45 (41–52)*	43 (40–47)	42 (39–47)	41 (39–47)	44 (41–49)	*45 (41–52)*
	End week	***16 (11–19)***	*7 (1–12)*	*7 (1–15)*	9 (5–16)	12 (10–16)	13 (10–16)	10 (6–15)	9 (4–13)
	Duration (weeks)	***30 (25–37)***	*14 (6–16)*	15 (13–18)	19 (17–24)	23 (21–25)	25 (22–27)	19 (16–21)	17 (14–19)
	Capture rate (%)	***95.0 (89.3–96.8)***	*66.1 (39.7–77.7)*	68.0 (58.2–76.6)	80.4 (73.9–92.5)	87.7 (84.8–92.5)	90.0 (87.4–94.6)	80.8 (78.0–87.4)	76.6 (75.9–77.3)
HHS 5	Start week	***46 (41–50)***	50 (46–3)	49 (45–1)	48 (44–52)	48 (44–52)	48 (44–51)	49 (46–1)	*51 (47–3)*
	End week	***18 (12–22)***	*11 (1–18)*	12 (7–15)	14 (9–18)	15 (13–18)	15 (13–18)	14 (9–16)	*11 (7–14)*
	Duration (weeks)	***24 (21–27)***	15 (6–21)	15 (12–18)	18 (16–22)	20 (17–23)	21 (18–23)	17 (15–18)	*13 (12–14)*
	Capture rate (%)	***95.5 (90–5.98.1)***	84.5 (30.0–92.8)	78.6 (71.0–86.6)	89.7 (83.8–94.5)	92.7 (90.2–94.0)	92.9 (91.9–94.5)	87.8 (80.2–90.1)	*76.1 (75.1–78.4)*
HHS 6	Start week	***41 (39–48)***	46 (43–52)	46 (43–51)	43 (42–48)	44 (42–48)	44 (42–48)	46 (43–50)	*47 (44–52)*
	End week	***15 (10–18)***	*8 (3–10)*	9 (3–13)	12 (3–15)	13 (10–15)	13 (10–15)	11 (9–14)	9 (4–11)
	Duration (weeks)	***26 (17–29)***	*14 (8–18)*	15 (13–18)	20 (14–23)	22 (17–24)	22 (17–24)	18 (15–19)	15 (10–16)
	Capture rate (%)	***95.1 (90.5–97.0)***	*76.7 (60.7–83.0)*	78.7 (67.0–84.9)	89.9 (71.3–94.9)	92.0 (90.4–94.9)	92.1 (90.4–94.9)	86.6 (81.1–91.9)	76.9 (75.3–78.2)
HHS 7	Start week	***46 (41–50)***	50 (48–4)	49 (47–1)	49 (44–51)	48 (42–51)	48 (41–50)	49 (45–1)	*51 (48–1)*
	End week	***17 (13–21)***	*11 (9–13)*	13 (10–15)	15 (11–17)	16 (12–20)	***17 (12–20)***	13 (11–17)	*11 (10–14)*
	Duration (weeks)	***24 (22–25)***	*12 (10–15)*	15 (14–19)	18 (17–22)	21 (19–25)	21 (20–25)	17 (16–19)	14 (13–16)
	Capture rate (%)	***94.5 (91.8–96.5)***	*73.1 (63.9–80.9)*	77.8 (73.0–86.4)	88.0 (85.3–91.8)	91.4 (89.9–95.8)	92.5 (91.0–95.8)	85.8 (83.6–88.9)	77.0 (75.8–78.1)
HHS 8	Start week	***51 (46–52)***	*3 (50–7)*	1 (51–4)	52 (49–4)	***51 (49–1)***	***51 (49–1)***	1 (50–3)	*3 (52–5)*
	End week	***18 (15–24)***	*13 (10–17)*	14 (13–21)	16 (13–24)	16 (13–21)	16 (13–21)	15 (12–19)	*13 (9–16)*
	Duration (weeks)	***22 (17–26)***	13 (4–16)	15 (13–18)	17 (15–24)	18 (16–22)	19 (16–22)	16 (14–17)	*11 (10–13)*
	Capture rate (%)	***95.8 (89.4–97.9)***	81.8 (40.3–93.5)	83.4 (78.3–87.9)	91.4 (83.5–94.2)	92.9 (91.4–97.9)	93.1 (91.4–97.9)	89.7 (83.5–91.7)	*76.2 (75.2–78.3)*
HHS 9	Start week	***47 (44–52)***	*1 (49–7)*	50 (48–3)	49 (46–52)	49 (46–51)	48 (46–51)	50 (48–1)	52 (50–2)
	End week	***15 (12–20)***	*9 (4–14)*	12 (11–17)	14 (11–16)	***15 (12–19)***	***15 (12–19)***	13 (11–16)	12 (9–16)
	Duration (weeks)	***21 (13–26)***	*11 (3–13)*	15 (14–17)	18 (16–19)	18 (17–24)	19 (17–24)	16 (14–17)	12 (11–15)
	Capture rate (%)	***93.3 (77.5–95.7)***	*68.1 (19.5–81.8)*	80.1 (70.7–88.0)	89.3 (80.9–93.0)	90.7 (86.3–95.9)	90.9 (86.3–95.9)	84.9 (79.1–90.0)	76.8 (75.0–78.2)
HHS 10	Start week	***47 (45–1)***	51 (50–3)	51 (49–1)	49 (46–1)	49 (48–1)	48 (46–1)	51 (48–1)	*52 (51–3)*
	End week	***17 (15–19)***	*11 (7–13)*	14 (9–15)	16 (14–16)	16 (12–19)	16 (14–21)	14 (12–17)	12 (10–15)
	Duration (weeks)	***23 (15–25)***	*12 (9–16)*	15 (12–17)	19 (15–23)	20 (15–21)	20 (15–23)	16 (15–19)	*12 (12–15)*
	Capture rate (%)	***95.3 (89.1–96.9)***	*74.6 (54.9–87.9)*	82.6 (65.4–87.2)	91.9 (86.0–96.2)	91.3 (89.1–94.4)	93.5 (89.1–95.2)	87.5 (85.9–89.1)	77.2 (75.7–78.3)

*Note:* In each region, we highlighted the earliest start and latest end weeks, longest duration, and highest capture rate (median values) in bold; and the latest start and earliest end weeks, shortest duration, and lowest capture rate (median values) in italics.

Most estimation methods produced a median capture rate above 80%, though the *10% positivity* method fell below this threshold in 8 of the 10 US HHS regions, as did the *MEM* in New Zealand, South Africa, and 6 of the 10 US HHS regions. The *75% AAP* method consistently yielded a median capture rate of just over 75% (ranging from 75.6 to 76.8%) due to its design (Tables 3 and 4). The lowest capture rates were observed in the US HHS regions using the 10% positivity method (56.8% in region 1, 65.4% in region 2, 66.1% in region 4, and 68.1% in region 9), and for the MEM in South Africa (59.7%). These three estimation methods typically marked the latest start, earliest end, and shortest RSV epidemic duration across all countries, with few exceptions. Among the other methods, differences in the median values of the four parameters were generally moderate. However, the *mean number* method tended to produce shorter RSV epidemics with lower capture rates (and thus, later start and earlier end weeks) compared to the *60% mean number* method, *mean positivity*, and *1.2% total detections* methods (Tables 3 and 4).

## 4 Discussion

In this study, we applied various estimation methods to RSV surveillance data from Chile, New Zealand, Singapore, South Africa, Spain, and the United States (over 441,0000 RSV cases overall) to evaluate how each method determined the start, end, duration, and capture rate of RSV epidemics. Our analysis revealed how the choice of method impacts the definitions of RSV transmission patterns. The 3% positivity method, for example, consistently identified an earlier start and longer duration of RSV epidemics compared to other methods. This ensured a higher capture rate but potentially triggered premature alerts and public health responses when RSV circulation was still low. Conversely, other real‐time methods such as the 10% positivity and MEM, as well as a retrospective method like AAP, typically delayed epidemic declarations, which could reduce the window for interventions.

Most estimations of the RSV epidemics we presented achieved capture rates above 80%, with some methods (such as mean positivity, 1.2% total detections, 60% mean number or mean number thresholds) yielding similar results. However, large differences were observed when comparing seasonality estimates in a country. For example, the MEM in South Africa resulted in a very low median capture rate of 59.7%: this is due to RSV circulating year‐round at modest levels in South Africa, with epidemic peaks that, in some seasons (e.g., 2012 in our series), stand out only slightly above the baseline level, or occasionally with the epidemic bridging consecutive years. Thus, while reflecting the unique seasonality of RSV circulation in the country, the results of the MEM for South Africa underscore the importance of carefully selecting methods based on specific public health objectives and context.

Although the 3% positivity method is useful for prospective monitoring, it could lead to premature or prolonged (and therefore, inefficient) use of healthcare resources during periods of low RSV impact. Thus, in settings prioritizing efficient resource allocation, an estimation method with shorter duration and high capture rate may be more suitable, as reflected in our study findings. A key aspect to note is that the epidemic duration defined by the 3% positivity rate method (as well as other estimation methods, but limited to South Africa and New Zealand) exceeds the 5‐month protective time window of Nirsevimab. This has critical and possible underappreciated implications for the organization of passive immunization programs, especially in those countries where administration is implemented in a seasonal rather than year‐round manner. Interestingly, the 10% positivity method achieved a very low capture rate in most US HHS regions due to its later start and earlier end of epidemics, a pattern less pronounced in other countries (except New Zealand, to a lesser extent). This discrepancy may reflect the lower RSV positivity rate in the United States, where applying a high threshold like 10% could delay epidemic detection, resulting in a shorter perceived duration of epidemics. Such differences may stem from regional RSV circulation patterns or variations of surveillance systems, case definitions, and testing strategies (e.g., lower positivity rates from PCR versus antigen‐based testing [16]). In general, it is crucial to consider that the performance of any method based on the positivity rate may be heavily impacted by the structure and characteristics of the surveillance system, e.g. whether it is primary care‐ or hospital‐based (or a mix of both), its reliance on voluntary reporting from both inpatient and outpatient settings (with less stringent criteria for sampling), the prioritization of viruses with a higher population burden (e.g., influenza or SARS‐CoV‐2), and whether it primarily targets vulnerable populations such as the elderly and children. Moreover, recommending the use of a fixed percent threshold across different settings might be problematic due to other factors that affect sensitivity and specificity, such as differences in age distribution and health seeking behaviors.

Among the estimation methods we applied, the MEM was the only one, aside from those based on positivity rate, that can be used as a real‐time (i.e., on a week‐by‐week basis) monitoring tool. However, it requires baseline data from several past seasons before it can be implemented. Retrospective methods (mean positivity rate, 1.2% total detections, mean number, 60% mean number, and 75% AAP), although generally not suitable for real‐time monitoring, are useful for descriptive and research purposes, such as cross‐country comparisons and trend analyses. With the exception of the 75% AAP method, these methods provided fairly comparable results.

A key limitation of our study is the small and somewhat homogenous sample of countries—five high‐income with “very high” human development index and one upper‐middle‐income country with “high” human development index [24, 25], which limits the geographical and epidemiological diversity in our dataset. The countries included in this study are generally well‐resourced for data collection; hence our findings may not fully reflect the patterns of RSV epidemics in lower‐resource settings. The demographic profile also showed a wide variation: the proportion of children aged 14 years and younger ranged from 14.3% in Singapore to 28.6% in South Africa, and that of the elderly aged 65 years and older ranged from 8.5% in South Africa to 19.2% in Spain [26]. Additionally, as mentioned in the methods, the surveillance systems of the countries included in this analysis differ in terms of in key features such as whether they are primary care‐ or hospital‐based, sentinel or non‐sentinel, the type of syndromic definition in use, and testing strategies. These differences (and others that might be more subtle and difficult to describe and quantify) may have introduced variability in the data that could affect the comparability of results, and whose impact is difficult to assess precisely. We relied on retrospective data from established surveillance systems which may not fully reflect real‐time challenges in public health decision‐making. The performance of methods based on positivity rate may have changed compared to the period covered by our investigation due to the emergence of SARS‐CoV‐2, a new, highly incident virus. This virus could potentially alter the expected positivity rate for other viruses that cause similar clinical symptoms. Lastly, we applied only some methods using a predefined set of rules, which, while widely used, do not capture all variations in estimation practices, such as the use of moving averages or minimum number of weeks for a threshold. Nevertheless, the selected methods are widely used in the literature [14], ensuring their relevance to current public health practices. In conclusion, we need to acknowledge that we have deliberately refrained from ranking the different estimation methods and recommending one over another, while aiming to highlight the strengths and intrinsic limitations of each of them. Rather, we emphasize that the choice of the method to be used for the estimation of the start, end, duration, and capture rate of RSV epidemics within pre‐defined seasons should be guided by the characteristics and performance of the surveillance system and the specific objectives of the monitoring effort. This guidance is relevant for both real‐time monitoring of epidemics, where the timing of an epidemic declaration has major implications for resource allocation and public health interventions. Future research in the field may assess the applicability of seasonality estimation methods in settings with less structured surveillance, further exploring how these methods align with clinical outcomes; comparative evaluations of their operational performance in real‐time scenarios, especially in the context of vaccine or mAb deployment, could help refine guidance for public health decision‐making.

## Author Contributions

**Lisa Staadegaard:** formal analysis, investigation, project administration, methodology, software, validation, visualization, writing – original draft. **Marco Del Riccio:** investigation, methodology, writing – original draft. **Susanne Heemskerk:** investigation, methodology, writing – review and editing. **Michel Dückers:** methodology, supervision, writing – review and editing. **Patricia Bustos:** investigation, resources, writing – review and editing. **Cheryl Cohen:** investigation, writing – review and editing, resources. **Jocelyn Moyes:** investigation, writing – review and editing, resources. **Vernon Jian Ming Lee:** investigation, writing – review and editing, resources. **Li Wei Ang:** investigation, writing – review and editing, resources. **Susana Monge:** investigation, writing – review and editing, resources. **Isabel Martínez‐Pino:** investigation, writing – review and editing, resources. **Mathieu Bangert:** funding acquisition, writing – review and editing, methodology. **Rolf Kramer:** funding acquisition, methodology, writing – review and editing. **John Paget:** conceptualization, funding acquisition, methodology, project administration, resources, supervision, writing – original draft. **Saverio Caini:** formal analysis, investigation, methodology, software, visualization, writing – original draft.

## Conflicts of Interest

M.B. and R.K. are Sanofi employees and may hold shares and/or stock options in the company. C.C. has received grant funds from US CDC and Taskforce for Global Health related to the current manuscript, and from BMGF and Sanofi Pasteur not related to the current manuscript. F.F.S., L.S., S.H., M.D., J.v.S., and S.C. report that Nivel has received RSV research grants from the Foundation for Influenza Epidemiology and Sanofi Pasteur. The remaining authors declare no competing interests.

### Peer Review

The peer review history for this article is available at https://www.webofscience.com/api/gateway/wos/peer‐review/10.1111/irv.70123.

## Supporting information


**FIGURE S1** HHS (US Department of Health and Human Services) regions, from: https://www.hhs.gov/about/agencies/iea/regional‐offices/index.html.
**FIGURE S2** Seasonality of RSV by country: 3‐week moving average number of detections in Chile, New Zealand, Singapore, South Africa, and Spain. Note: the *y*‐axis scale differs between countries, to accommodate the widely varying numbers of RSV detections by country.
**FIGURE S3** Seasonality of RSV by country: 3‐week moving average number of detections in the United States by HHS region. Note: the *y*‐axis scale differs between HHS regions, to accommodate the widely varying numbers of RSV detections by country.

## Data Availability

The data that support the findings of this study are available from the corresponding author upon reasonable request.
