# Supplementary material for: Evaluating the Impact of Different Methods on the Timing and Duration of RSV Epidemics: Analysis of Surveillance Data From the GERi (Global Epidemiology of RSV in Hospitalized and Community Care) Study
Source: Influenza Other Respir Viruses. 2025 Jun 1;19(6):e70123. doi: 10.1111/irv.70123 (PMC12127213; doi:10.1111/irv.70123)
Supplement: Supplementary file 1 — FIGURE S1 HHS (US Department of Health and Human Services) regions, from: https://www.hhs.gov/about/agencies/iea/regional‐offices/index.html. FIGURE S2 Seasonality of RSV by country: 3‐week moving average number of detections in Chile, New Zealand, Singapore, South Africa, and Spain. Note: the y‐axis scale differs between countries, to accommodate the widely varying numbers of RSV detections by country. FIGURE S3 Seasonality of RSV by country: 3‐week moving average number of detections in the United States by HHS region. Note: the y‐axis scale differs between HHS regions, to accommodate the widely varying numbers of RSV detections by country. [file IRV-19-e70123-s001.docx]

**Supplementary Figure 1**. HHS (U.S. Department of Health and Human Services) regions, from: <https://www.hhs.gov/about/agencies/iea/regional-offices/index.html>


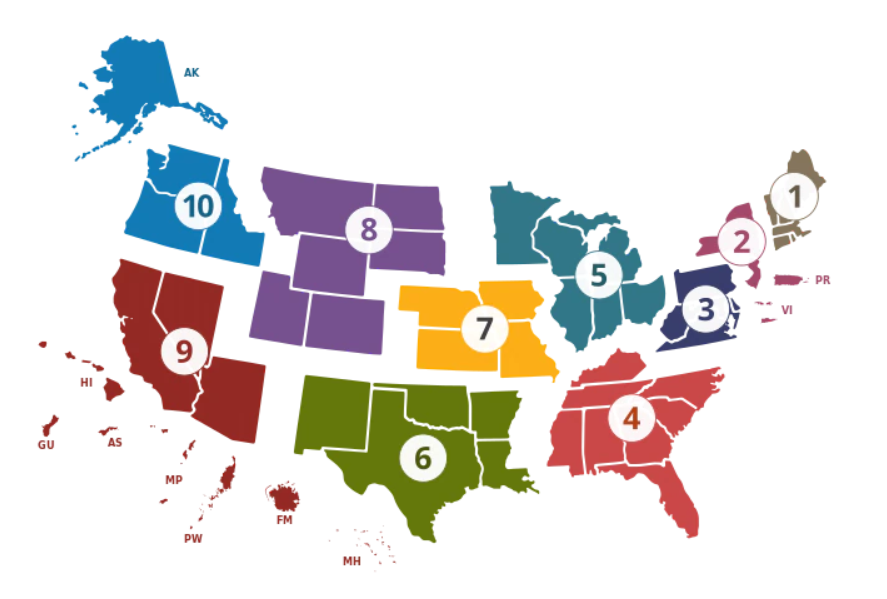


**Supplementary Figure 2.** Seasonality of RSV by country: 3-week moving average number of detections in Chile, New Zealand, Singapore, South Africa and Spain. Note: the y-axis scale differs between countries, to accommodate the widely varying numbers of RSV detections by country.

******

**Supplementary Figure 3.** Seasonality of RSV by country: 3-week moving average number of detections in the USA by HHS region. Note: the y-axis scale differs between HHS regions, to accommodate the widely varying numbers of RSV detections by country.
